# Shear wave elastography for quantitative assessment of nerve tissue stiffness after sciatic nerve decompression in diabetic rats

**DOI:** 10.3389/fneur.2026.1962826

**Published:** 2026-10-23

**Authors:** Dian-Cheng Li, Wen-Xue Li, Yu-Wei Xin, Dan-Dan Li, Jia-An Zhu

**Affiliations:** Department of Ultrasound, Peking University People’s Hospital, Beijing, China

**Keywords:** diabetes mellitus, diabetic peripheral neuropathy, nerve decompression, rat, shear wave elastography

## Abstract

**Introduction:**

Diabetic peripheral neuropathy (DPN) is a common complication of diabetes mellitus. Surgical decompression of distal DPN has been shown to reduce ulceration and amputation rates, though recurrence risk persists, warranting postoperative follow-up. Current DPN evaluation relies on clinical symptoms, signs, and nerve conduction studies (NCS), which have inherent limitations. Shear wave elastography (SWE) is a non-invasive, cost-effective technique that quantifies tissue stiffness and reflects underlying pathology, and has been reported as a potential tool for early detection of diabetic neuropathy. This study aimed to assess the value of SWE for quantitative evaluation of nerve stiffness after sciatic nerve decompression in diabetic rats, providing a preliminary theoretical basis for future clinical research.

**Methods:**

A total of 24 male Sprague–Dawley (SD) rats were used in this study. Six served as normal controls (*n* = 6). The remaining 18 underwent sciatic nerve compression to establish a diabetic neuropathy model. Four weeks later, 6 of these were assigned to the diabetic sciatic nerve compression 4-week group (DSNC, *n* = 6), while the other 12 received surgical decompression and were further divided into 2-week (DSND2, *n* = 6) and 8-week (DSND8, *n* = 6) post-decompression groups. Nerve stiffness was assessed by SWE, alongside motor nerve conduction velocity (MNCV) and histopathological examination.

**Results:**

In the DSND2 group, sciatic nerve stiffness was significantly decreased compared with the DSNC group (*p* < 0.01), with a further decrease in the DSND8 group (*p* < 0.05 vs. DSND2). No significant differences in MNCV, myelinated fiber density, myelin sheath thickness, or average axon diameter were found between the DSND2 and DSNC groups (*p* > 0.05). In contrast, the DSND8 group showed significant improvements in all these parameters versus the DSNC group (*p* < 0.05), with MNCV reaching 49.17 ± 4.71 m/s. Moreover, myelinated fiber density and myelin sheath thickness were also significantly higher in the DSND8 group than in the DSND2 group (*p* < 0.05).

**Conclusion:**

Dynamic SWE assessment of decreased neural stiffness after sciatic nerve decompression predicts functional improvement in diabetic rats, and may serve as a noninvasive evaluation method.

## 1 Introduction

Diabetic peripheral neuropathy (DPN) is one of the leading causes of chronic ulcers, gangrene, and amputation of the lower extremities (1). It has been documented that up to 50% of individuals with diabetes mellitus (DM) experience painful manifestations attributable to diabetic neuropathy, which not only diminishes their quality of life but also imposes a substantial economic burden (2).

DPN is defined as a symmetrical, length-dependent sensorimotor polyneuropathy attributable to diabetes mellitus, occurring in the absence of other discernible etiologies of peripheral nerve dysfunction (3). Given that the natural history of DPN is generally progressive and irreversible, tight glucose control strategies have yielded conflicting preventive outcomes, and standard pharmacotherapies, while often providing symptomatic analgesia, do not arrest neurological deterioration (4, 5). The prevalence of carpal tunnel syndrome (CTS) in DM patients is as high as approximately 30% (6). Additionally, nerve entrapment has been demonstrated to accelerate the progression of diabetic foot syndrome (7). The “double crush” hypothesis is widely considered to be a major contributing factor to the increased susceptibility to nerve entrapment in DM (8). Dellon was the first to introduce nerve decompression surgery for the treatment of DPN affecting the distal limbs, and demonstrated its effectiveness in reducing the risk of ulceration and amputation (9, 10). Nevertheless, the risk of ulcer recurrence remains after surgical decompression for DPN (11). Postoperative follow-up is still required.

At present, clinical scoring instruments and nerve conduction studies (NCS) are extensively utilized in the assessment of diabetic peripheral neuropathy (12), among which NCS is considered gold standard method (13). However, clinical scores are inherently subjective, and NCS are time-consuming and associated with patient discomfort (14). Moreover, DM patients with CTS undergo nerve decompression surgery 4 to 14 times more frequently than the general population (15). Therefore, a novel complementary assessment method that enables repeated evaluation of postoperative neurological recovery is urgently needed.

Shear wave elastography (SWE) can quantitatively measure tissue stiffness, thereby reflecting underlying pathological changes (16). SWE has been widely applied in various organs, such as liver (17), breast (18), and thyroid (19). Shear-wave velocity (SWV) has been reported to correlate positively with tissue stiffness (20). In recent years, SWE has emerged as a valuable modality for the assessment of diabetic peripheral neuropathy, with accumulating evidence indicating that peripheral nerve stiffness is substantially elevated in patients with DPN as compared to both diabetic patients without neuropathy and healthy control subjects (21, 22). However, the value of SWE for the assessment of diabetic peripheral neuropathy following decompression surgery remains unclear. Furthermore, the association between SWE-derived nerve stiffness and histopathological alterations after surgical decompression for DPN has yet to be established.

Given these deficiencies in research, this study aimed to evaluate the utility of SWE in the assessment of diabetic rats following nerve decompression, and to further examine the correlation between SWE-measured nerve stiffness and the histopathological as well as functional alterations of the sciatic nerve.

## 2 Method

### 2.1 Animals and groups

A total of 24 male Sprague–Dawley (SD) rats (Beijing Vital River Laboratory Animal Technologies Co. Ltd., China) aged 8 weeks and weighing 250–300 g were used in this study; 6 normal SD rats were selected as the control group (*n* = 6), and 18 SD rats were included in the diabetic group and subjected to sciatic nerve compression modeling. Four weeks later, 6 rats were randomly designated as the diabetic sciatic nerve compression 4-week group (DSNC, *n* = 6), whereas the other 12 rats received surgical decompression of the sciatic nerve. According to the different periods after decompression, 12 rats were further randomly subdivided into diabetic sciatic nerve decompression 2-week group (DSND2, *n* = 6) and 8-week group (DSND8, *n* = 6). All experimental procedures involving animals were approved by the Institutional Animal Care and Use Committee of Peking University People’s Hospital (ethics number: 2017PHC061).

### 2.2 Sciatic nerve compression modeling in diabetic rats

All SD rats were housed in a specific pathogen-free facility for a 7-day acclimatization period prior to experimentation. Diabetes was induced by feeding animals a high-glucose and high-fat diet for 4 weeks, followed by a single intraperitoneal injection of streptozotocin (STZ, 40 mg/kg body weight, Sigma Chemical Co., USA). Diabetes status was confirmed 7 days after STZ administration by measuring random blood glucose levels, with values exceeding 16.7 mmol/L considered indicative of successful diabetes induction (23). Blood samples(0.4 mL)were obtained from the tail vein of the rats. For surgical procedures, anesthesia was achieved via intraperitoneal administration of ketamine at a dosage of 60 mg/kg. Following anesthesia, the left hind limb sciatic nerve was surgically exposed. A 1-cm-long silicone tube with an internal diameter of 1.2 mm was then placed around the sciatic nerve at the middle third level (24). The tube was longitudinally slit to facilitate atraumatic placement around the nerve using fine dissecting scissors, allowing the nerve to be gently encircled with minimal manipulation. The tube was secured in position with 6–0 nylon sutures to prevent displacement along the nerve axis. All surgical interventions were carried out by a single investigator to eliminate inter-operator variability, and strict aseptic technique was maintained throughout all procedures.

### 2.3 Sciatic nerve decompression in diabetic rats

At 4 weeks after sciatic nerve compression, rats were anesthetized with an intraperitoneal injection of ketamine (60 mg/kg), after which the silicone tube was removed in the decompression group under meticulous manipulation to avoid iatrogenic nerve injury. Adjacent inflammatory tissue was debrided, and the epineurium was carefully released. All procedures were performed by a single orthopedic surgeon experienced in peripheral nerve microsurgery.

### 2.4 SWE assessment of the sciatic nerve

SWE acquisitions were performed using a 20 MHz linear-array transducer (SuperSonic Imagine, France). Prior to imaging, rats received an intraperitoneal injection of ketamine (60 mg/kg) for anesthesia. The silicone tube and adjacent inflammatory tissues encasing the sciatic nerve were carefully dissected away to preserve neural integrity. Rats were placed in the lateral decubitus position with the hind limb relaxed, and longitudinal nerve images were acquired, with care taken to avoid transducer-induced compression. The ROI was set at 0.01 cm^2^ and positioned within the epineurium. The SWE of nerves was measured 3 times at the compression site to increase reproducibility, with the average value being chosen for the following analysis. Two experienced operators (over 5 years experiences in ultrasound examination) independently evaluated all groups; the second operator repeated the measurements using the same protocol while blinded to the first operator’s results.

### 2.5 Electrophysiological testing of sciatic nerve

Rats were received ketamine anesthesia via intraperitoneal administration at a dosage of 60 mg/kg. Serial measurements of motor nerve conduction velocity (MNCV) in the sciatic nerve were performed using a TECA Synergy electrophysiological system (VIASYS Healthcare, USA) at 4 weeks after nerve compression (DSNC group), as well as at 2 weeks (DSND2 group) and 8 weeks (DSND8 group) after decompression in diabetic rats and control group. Bipolar stimulating electrodes were positioned proximally and distally to the compressed segment of the sciatic nerve, with stimulation parameters set as follows: pulse duration of 0.1 ms, frequency of 1 Hz, and intensity of 0.12 mA. The reference electrode was implanted into the quadriceps femoris muscle, while the active recording electrode was secured over the tibialis anterior muscle for signal acquisition.

### 2.6 Histomorphometric analysis of the sciatic nerve

Sciatic nerve tissues(10 mm)were initially immersed overnight at 4 °C in 3% glutaraldehyde prepared in 0.1 M phosphate buffer (PB, pH 7.4), followed by a 2-h secondary fixation in 1% osmium tetroxide (OsO₄; Sigma Chemicals Co., USA) dissolved in PB. The samples were then subjected to graded ethanol dehydration and transitional treatment with propylene oxide (Electron Microscopy Sciences, Hatfield, PA, USA), subsequently embedded in Durcupan ACM resin (Fluka, Switzerland), and cured at 60 °C. Semithin cross-sections (1 μm) were cut on an ultramicrotome (MT 6000-XL, RMC, Tucson, AZ) and stained with 1% toluidine blue (Merck, Germany) in 1% sodium tetraborate (Ecibra, Brazil). Light microscopy (Olympus BX51, Japan) at ×1,000 magnification was used to examine these sections for quantifying myelinated fiber density (fibers per mm^2^). Thereafter, ultrathin sections (50 nm) were prepared from the resin-embedded blocks, counterstained with uranyl acetate and lead citrate, and, following dehydration with ethanol and acetone, visualized under a transmission electron microscope (Fei Tecnai G2 F20, USA) at ×2,550 magnification. This enabled measurement of myelin sheath thickness (μm) and average axon diameter. All morphometric quantitative assessments were performed using Image-Pro Plus 6.0 software, following established protocols (25, 26). A single examiner, masked to group allocation, carried out all measurements.

### 2.7 Statistical studies

Statistical analyses were conducted using SPSS version 22.0. All data are expressed as means ± standard deviations. Normality of data distribution was assessed using the Anderson–Darling test. For intergroup comparisons, one-way analysis of variance (ANOVA) was applied, followed by either the Bonferroni *post hoc* test or Tamhane’s T2 test, depending on the equality of variances. The association between SWE values and MNCV was evaluated using Pearson’s correlation coefficient. Interobserver reliability for SWE measurements was quantified via the intraclass correlation coefficient (ICC).

## 3 Result

### 3.1 SWE findings before and after sciatic nerve decompression in diabetic rats

The mean values of sciatic nerve stiffness evaluated by SWE in each group of rats are presented in Table 1. Representative SWE images of each group are shown in Figure 1. Sciatic nerve tissue stiffness in the DSNC group was significantly increased compared with the control group (*p* < 0.01). In the DSND2 group, sciatic nerve stiffness decreased markedly relative to the DSNC group (*p* < 0.01), with a further reduction observed in the DSND8 group compared with the DSND2 group (*p* < 0.05), indicating a dynamic and progressive decrease in sciatic nerve stiffness. However, the values of sciatic nerve stiffness in the DSND8 group remained significantly higher than those in the normal control group (*p* < 0.05). There was excellent interobserver consistency of SWE. The consistency value for the elasticity was 0.862.

**Table 1:** Data of SWE.

Operator	Groups	SWV(m/s) (mean ± SD)	95% CI	
			Lower	Upper
Operator 1	CON	1.75 ± 0.05	1.7	1.81
	DSNC	2.48 ± 0.11*	2.36	2.59
	DSND2	2.09 ± 0.13*	1.95	2.22
	DSND8	1.93 ± 0.09*	1.83	2.02
Operator 2	CON	1.77 ± 0.06	1.71	1.83
	DSNC	2.45 ± 0.05*	2.4	2.5
	DSND2	2.04 ± 0.11*	1.93	2.16
	DSND8	1.91 ± 0.09*	1.81	2

SWV, shear wave velocity; CON, control group; DSNC, sciatic nerve after 4-week compression in the diabetic rats; DSND2, sciatic nerve after 2-week decompression in the diabetic rats; DSND8W, sciatic nerve after 8-week decompression in the diabetic rats. *p < 0.05 when compared cross groups.

**Figure 1:**
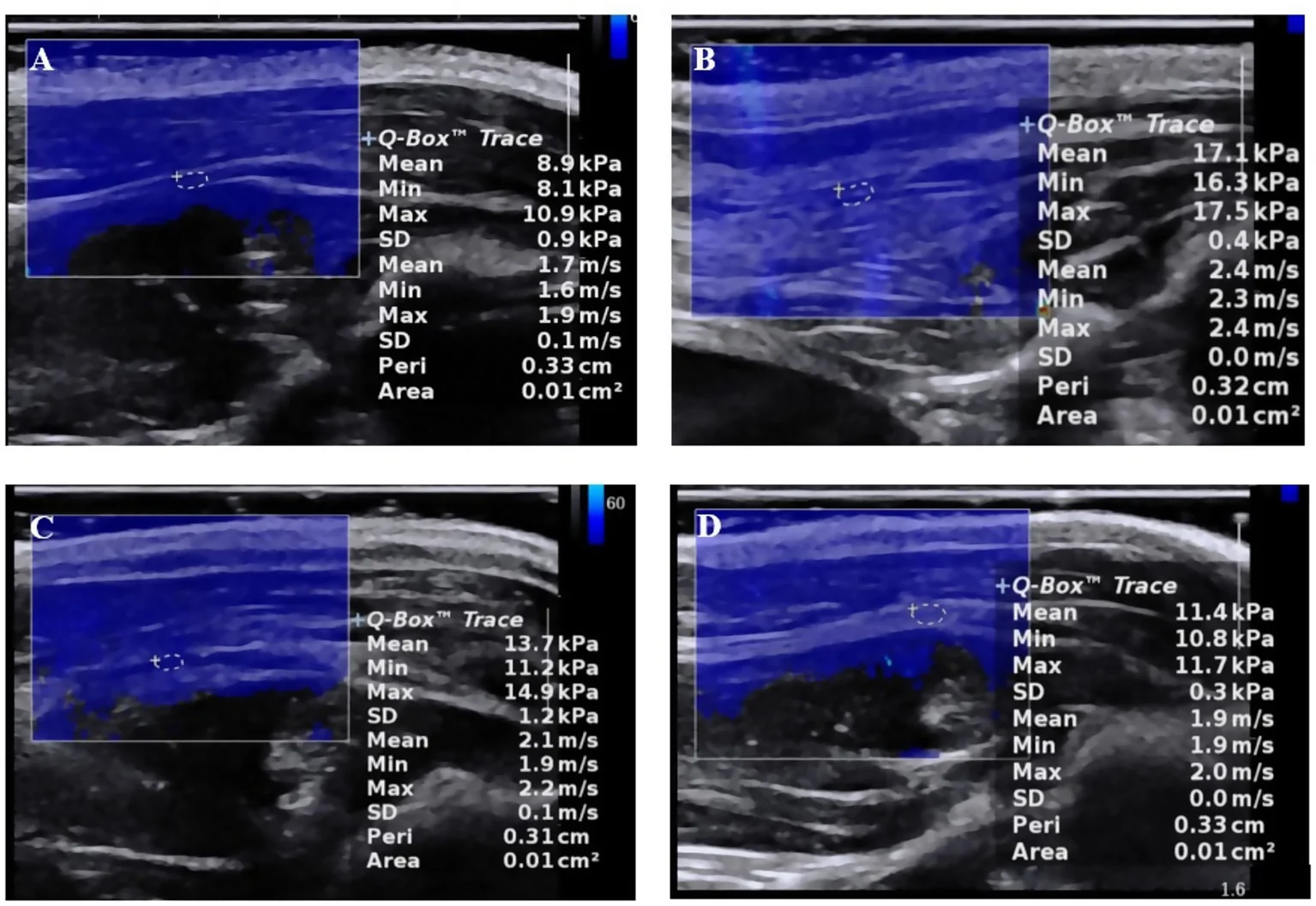
Schematic representation of SWE of the sciatic nerve in rats. **(A)** SWE image of the sciatic nerve in the control group(CON). **(B)** SWE image of the sciatic nerve in the DSNC group. **(C)** SWE image of the sciatic nerve in the DSND2 group. **(D)** SWE image of the sciatic nerve in the DSND8 group.

### 3.2 Pre- and post-decompression MNCV of the sciatic nerve

The MNCV in the DSND2 group was 40.83 ± 4.67 m/s, which did not differ significantly from that in the DSNC group (35.83 ± 2.93 m/s) (*p* > 0.05). In contrast, the MNCV in the DSND8 group was 49.17 ± 4.71 m/s, which was significantly improved compared with the DSNC group (*p* < 0.05); however, it remained substantially lower than that in the normal control group (65.5 ± 5.43 m/s) (*p* < 0.05)(Figure 2).

**Figure 2:**
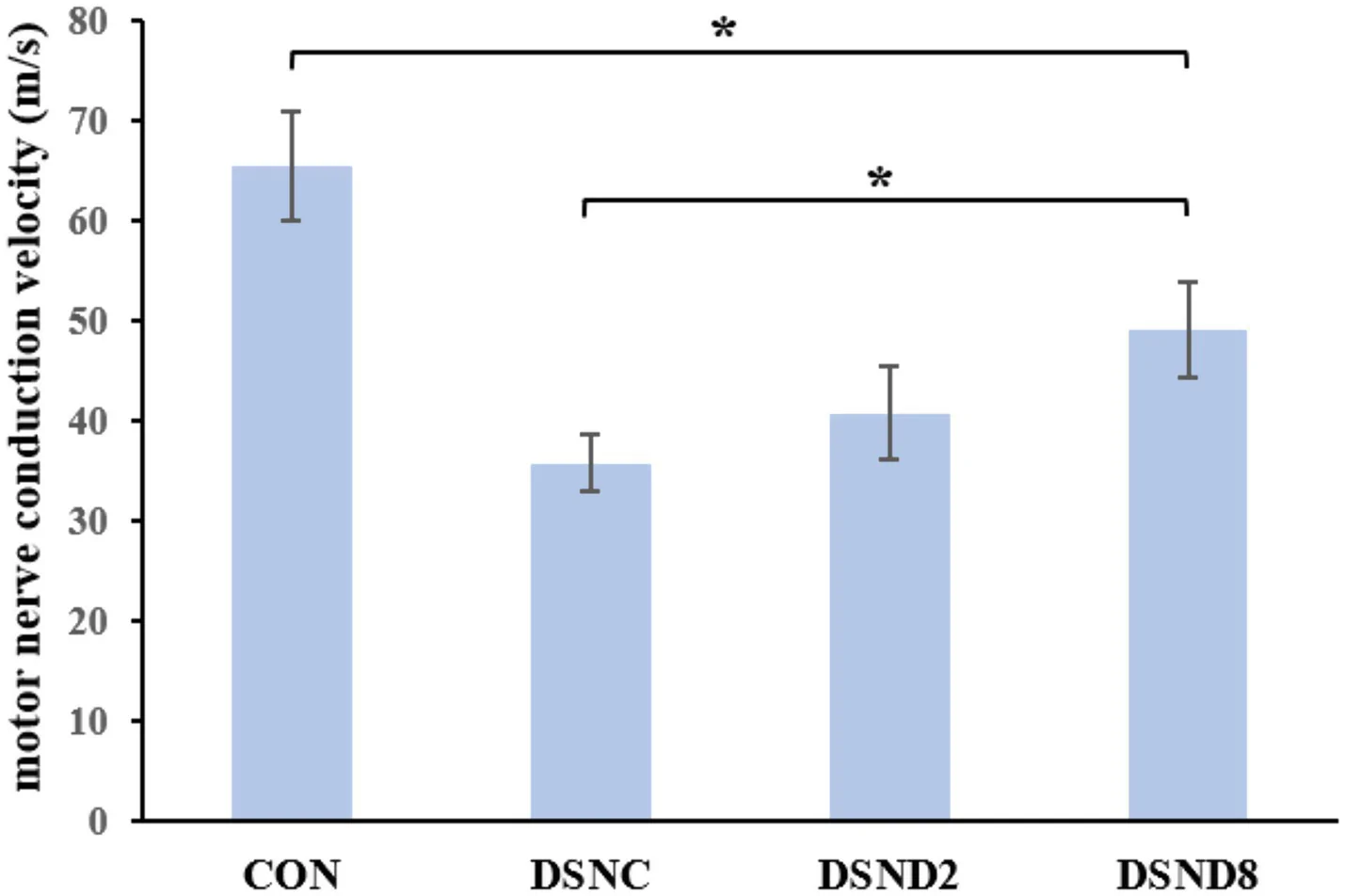
Alterations in motor nerve conduction velocity in diabetic rats. CON, control group; DSNC, sciatic nerve after 4-week compression in the diabetic rats; DSND2, sciatic nerve after 2-week decompression in the diabetic rats; DSND8W, sciatic nerve after 8-week decompression in the diabetic rats. *p < 0.05.

### 3.3 Semi-quantitative histomorphological findings

Histomorphological analysis revealed that myelinated fiber density was significantly lower in the DSNC group (6,522 ± 476 vs. 12,780 ± 1,202 fibers/mm^2^ in control group, *p* < 0.05). No significant difference was detected between the DSND2 (7,979 ± 687 fibers/mm^2^) and DSNC groups (*p* > 0.05), while a marked increase was observed in the DSND8 group (11,008 ± 1,413 fibers/mm^2^) compared with DSND2 (*p* < 0.05) (Figures 3, 4). Similarly, average axon diameter and myelin thickness in the DSND2 group (4.36 ± 1.50 μm and 0.83 ± 0.18 μm) did not differ from those in the DSNC group (3.89 ± 0.96 μm and 0.84 ± 0.17 μm) (*p* > 0.05), whereas both measures in the DSND8 group (4.71 ± 0.93 μm and 0.99 ± 0.22 μm) were significantly greater than in DSNC (*p* < 0.05), but remained inferior to control group (*p* < 0.05) (Figure 4).

**Figure 3:**
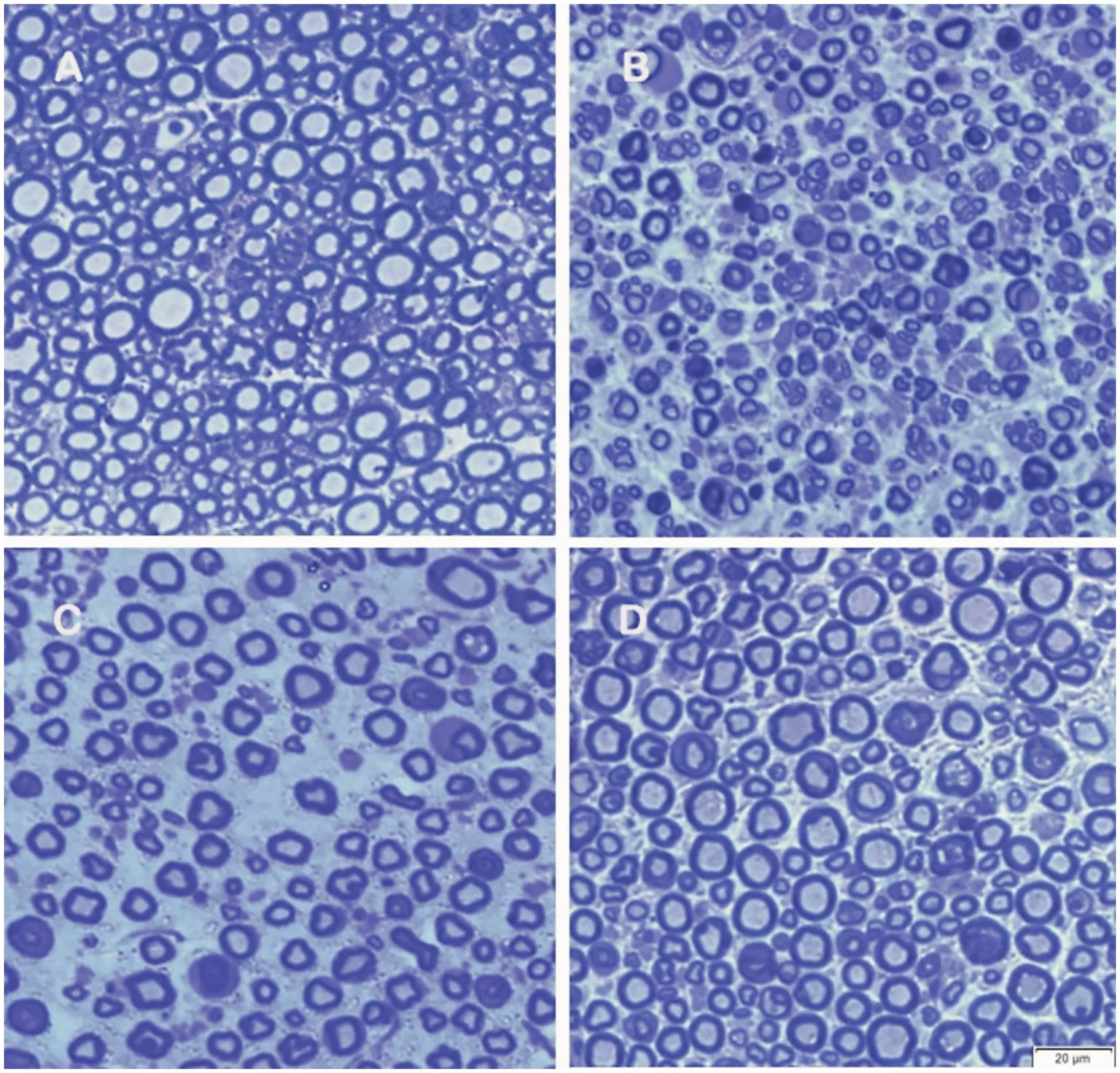
Toluidine blue-stained semithin sections of the sciatic nerve. **(A)** The control group (CON). **(B)** Sciatic nerve compression 4-week group in the diabetic rats (DSNC). **(C)** Sciatic nerve decompression 2-week group in the diabetic rats (DSND2). **(D)** Sciatic nerve decompression 8-week group in the diabetic rats (DSND8). (Magnification: ×400; scale bar 20 μm).

**Figure 4:**
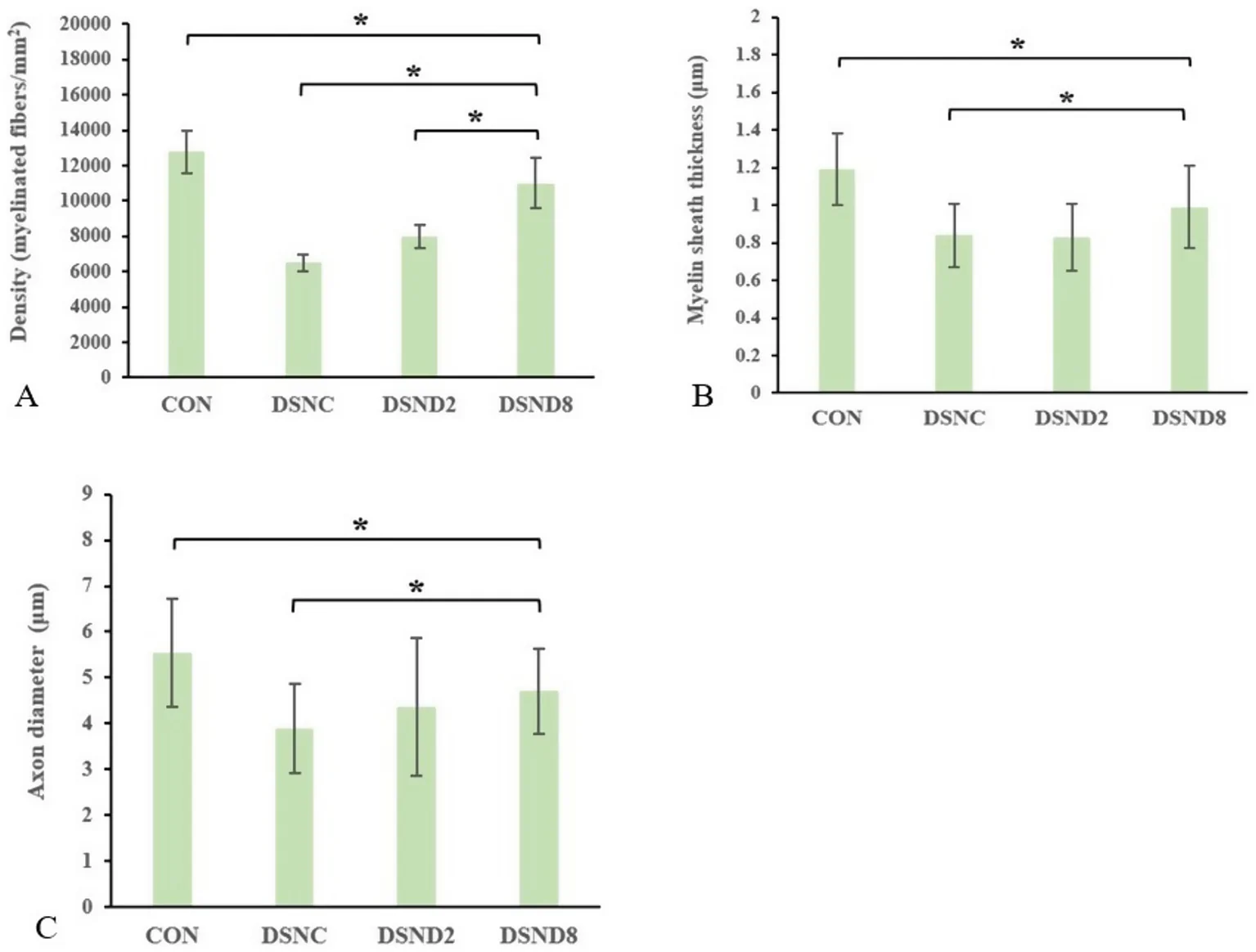
Morphometric parameters of the sciatic nerve fibers. All groups of sciatic nerve morphometric parameters are shown as density of myelinated fibers **(A)**, myelin sheath thickness **(B)**, and axon diameter **(C)**. CON, control group; DSNC, sciatic nerve after 4-week compression in the diabetic rats; DSND2, sciatic nerve after 2-week decompression in the diabetic rats; DSND8W, sciatic nerve after 8-week decompression in the diabetic rats.*p < 0.05.

## 4 Discussion

This study was based on the concept of “double crush” in DPN and employed the model of sciatic nerve compression using a silicone tube in STZ-induced diabetic rats, as originally described by Dellon, a widely used approach for investigating peripheral nerve entrapment in the context of diabetes (24). Given that an extended duration of compression may result in substantial nerve injury and thus affect the experimental outcomes, the 4-week time point was chosen for decompression (27, 28). In this study, a significant decrease in MNCV was observed at 4 weeks post-compression, along with notable reductions in myelinated fiber density, axon diameter, and myelin sheath thickness, all of which are indicative of severe sciatic nerve damage.

Consistent with previous reports (29), no significant improvement in MNCV, myelinated fiber density, axon diameter, or myelin thickness was observed at 2 weeks after surgery compared with preoperative condition, indicating negligible functional recovery. Pettersson et al. (28) found that despite functional improvement at 8 weeks post-decompression, degenerative changes in the nerve were still present. Meanwhile, Wang et al. (30) demonstrated that in STZ-induced diabetic rats undergoing surgical decompression at 4 weeks following sciatic nerve compression, significant improvements in nerve conduction velocity, latency, and amplitude, as well as an increase in myelinated fiber diameter, were observed at 8 weeks after decompression, indicative of restored sciatic nerve function. Accordingly, 8 weeks post-decompression was selected as the second time point for longitudinal observation in this study.

The results demonstrated that at 8 weeks post-decompression, MNCV, myelinated fiber density, axonal diameter, and myelin sheath thickness were all significantly increased compared with those at 2 weeks post-decompression, indicating a dynamic process of neurological functional recovery. However, none of these parameters had returned to normal condition. In agreement with our present study, several studies have reported that electrophysiological and histomorphological data of the sciatic nerve in diabetic rats did not fully return to normal condition at 8 weeks following nerve decompression surgery (29, 30). The underlying mechanisms of DPN are multifactorial: hyperglycemia drives mitochondrial reactive oxygen species overproduction, inflammation, and endoneurial edema (31); dyslipidemia exacerbates neural injury via free fatty acid-induced inflammatory signaling (32); and metabolic disturbances disrupt endoplasmic reticulum proteostasis and calcium homeostasis, triggering neuronal apoptosis (33). These multifactorial pathophysiological processes may account for the incomplete recovery observed at 8 weeks post-decompression; nevertheless, marked improvements were still evident compared with the pre-decompression baseline.

SWE has been applied to evaluate peripheral nerve entrapment, such as carpal tunnel syndrome (34) and ulnar tunnel syndrome (35), in which nerve stiffness is higher than that of healthy tissue. In addition, SWE has been reported to be a potential tool for the early detection and diagnosis of diabetic neuropathy, as it shows increased stiffness in affected nerves (36). However, whether decreased neural stiffness after decompression predicts regression of pathology in diabetic peripheral neuropathy remains unclear.

In our study, sciatic nerve tissue stiffness decreased at 2 weeks post-decompression. This result is consistent with the findings of Wang et al. (37). But, neither histopathological nor electrophysiological findings indicated recovery of nerve function. The decrease in neural tissue stiffness at this stage likely results from the release of pressure following decompression surgery (38). Yoshii et al. (39), using strain elastography, found that nerve stiffness decreased at 3 months after carpal tunnel release, accompanied by improvements in nerve conduction velocity and clinical symptoms. Similarly, this study found neural tissue stiffness further decreased at 8 weeks post-decompression, and both histomorphological and electrophysiological findings indicated improvement in nerve function. Nerve decompression reduced neural edema (40), improved microcirculation and regenerative capacity, and partially reversed the underlying pathological process in the sciatic nerve of diabetic rats (41). Furthermore, our study went a step further to show that the progressive decrease in neural tissue stiffness after nerve decompression correlated with functional recovery of the sciatic nerve in diabetic rats.

There are several limitations in this study: (a) The present study was a preclinical animal-based investigation with a relatively limited sample size. (b) This study was limited to an 8-week postoperative follow-up; consequently, the long-term benefit of nerve decompression for DPN remains indeterminate. Further investigations with extended observation periods are needed to be investigated in future study. (c) The use of STZ-induced diabetic rats without glycemic management in this study does not completely mirror clinical conditions. (d) In this study, gait analysis, mechanical pain threshold, and thermal pain threshold were not assessed in diabetic rats after sciatic nerve decompression. The inclusion of these behavioral parameters in future studies would strengthen the evidence.

## 5 Conclusion

Dynamic SWE assessment of progressive decrease in neural stiffness after sciatic nerve decompression predicts postoperative functional improvement in diabetic rats. Thus, SWE may be used as a noninvasive and effective evaluation method following diabetic peripheral nerve decompression.

## Data Availability

The original contributions presented in the study are included in the article/supplementary material, further inquiries can be directed to the corresponding author.
